# Essential functions of miR‐125b in cancer

**DOI:** 10.1111/cpr.12913

**Published:** 2020-12-17

**Authors:** Boya Peng, Poh Ying Theng, Minh T. N. Le

**Affiliations:** ^1^ Department of Pharmacology Yong Loo Lin School of Medicine National University of Singapore Singapore Singapore; ^2^ Department of Biomedical Sciences School of Veterinary Medicine and Life Sciences City University of Hong Kong Kowloon Hong Kong; ^3^ N.1 Institute for Health National University of Singapore Singapore Singapore; ^4^ City University of Hong Kong Shenzhen Research Institute Shenzhen China

**Keywords:** biomarker, cancer, chemoresistance, microRNA, miR‐125b

## Abstract

MicroRNAs (miRNAs) are small and highly conserved non‐coding RNAs that silence target mRNAs, and compelling evidence suggests that they play an essential role in the pathogenesis of human diseases, especially cancer. miR‐125b, which is the mammalian orthologue of the first discovered miRNA *lin‐4* in *Caenorhabditis elegans*, is one of the most important miRNAs that regulate various physiological and pathological processes. The role of miR‐125b in many types of cancer has been well established, and so here we review the current knowledge of how miR‐125b is deregulated in different types of cancer; its oncogenic and/or tumour‐suppressive roles in tumourigenesis and cancer progression; and its regulation with regard to treatment response, all of which are underlined in multiple studies. The emerging information that elucidates the essential functions of miR‐125b might help support its potentiality as a diagnostic and prognostic biomarker as well as an effective therapeutic tool against cancer.

## Graphical abstract

miR‐125b is an important microRNA in cancer. It is deregulated in different types of cancer where it acts as an oncogene and/or a tumour suppressor in a context‐dependent manner. miR‐125b interacts with multiple upstream regulators and downstream targets, which are involved in a variety of signalling pathways and cellular processes during tumourigenesis and cancer progression. It also exerts as a key regulator with regards to the response of cancer cells to chemotherapy. miR‐125b holds a great potential in clinical cancer diagnosis, prognosis and therapies. *Created with* BioRender.com.

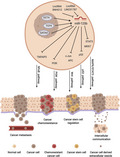

## 1 INTRODUCTION

### 1.1 MicroRNAs

Research conducted over the last two decades has identified microRNAs (miRNAs) as an important class of endogenous gene expression regulators in all tissues.^1^ *Cel‐lin‐4* is the first discovered miRNA in *Caenorhabditis elegans* in 1993.^2^ It is known to be essential for the temporal control of post‐embryonic proliferation and differentiation, timing of neuronal rewiring, vulva formation, dauer larva formation and the terminal differentiation of hypodermal cells.^2^, ^3^, ^4^, ^5^, ^6^, ^7^ miRNAs are small, evolutionarily conserved non‐coding RNAs of on average ~22 nucleotides in length, and they mediate transcriptional and post‐transcriptional gene regulation through translational repression or transcript cleavage in a sequence‐dependent manner. With the help of the enzyme Dicer and protein complexes, miRNAs remodel chromatin, cleave or degrade targeting mRNAs and block protein synthesis.^8^, ^9^, ^10^ Unlike siRNAs and shRNAs, which are perfectly complementary to their target mRNAs, an miRNA typically binds to one or more partially complementary mRNAs at their 3′ untranslated region (UTR), or (in some cases) at their 5′ UTR, gene promoters and coding sequences.^8^, ^11^ However, the seed region (ie nucleotides at positions 2‐7) of the miRNA must be a perfect match for its targets.^8^ Dysregulation of an miRNA might affect the expression of various genes by binding to multiple sites with the same seed matches. In this way, they are critical for a variety of biological processes, such as metabolic homeostasis, cell proliferation, apoptosis and differentiation.^12^, ^13^, ^14^

In the past decade, various studies have elucidated that miRNAs are tightly associated with the pathogenesis of human disease, especially cancer.^15^, ^16^, ^17^ miRNAs function either as tumour suppressors by negatively regulating oncogenic targets or as oncogenes (also called oncomiRs) through down‐regulating tumour‐suppressive target mRNAs.^18^ As tumour suppressors, miRNAs can be re‐introduced into cancer cells by using miRNA mimics to inhibit tumour development. For example, the expression of miR‐16 is down‐regulated in prostate cancer.^19^ In a prostate cancer mouse xenograft model, the systematic delivery of miR‐16 mimics has been shown to suppress tumour progression and inhibit metastasis.^19^ miR‐22 is another down‐regulated tumour suppressor, which has been identified in breast cancer.^20^ The intra‐tumoural delivery of miR‐22 mimics inhibits the growth of breast tumour by inducing cellular senescence.^20^ There are also many examples of miRNAs acting as oncogenes (oncomiRs) in tumourigenesis. In colorectal cancer, for example, an up‐regulation of miR‐32 is associated with an enhanced level of cancer cell differentiation and tumourigenesis by promoting its proliferative and metastatic abilities.^21^ In addition, in ovarian cancer, an up‐regulation of miR‐181a results in reduced cell apoptosis and enhanced epithelial‐mesenchymal transition (EMT).^22^ Recently, anti‐miRNA therapeutics have attracted the attention of many biopharmaceutical companies.^23^ Silencing oncogenic miRNAs is a rational strategy to suppress miRNA‐mediated tumourigenesis. Experimentally, oncogenic miRNA expression can be repressed by lentivirally delivered shRNAs or ‘sponges’ containing miRNA‐binding sites.^23^ However, lentiviral vectors are not suitable for clinical application due to the risk of insertional mutagenesis.^23^ Therefore, most anti‐miRNA therapeutics are currently being developed based on synthetic antisense oligonucleotides (ASOs), which are complementary to the miRNAs.^23^ Indeed, several clinical trials for miRNA ASOs are currently underway, including one for anti‐miR‐380 ASOs to treat neuroblastoma and one for anti‐miR‐122 ASOs to treat hepatitis C.^23^ To date, several pre‐clinical and clinical trials have already been carried out based on miRNA therapeutics.^9^

### 1.2 MiR‐125b cluster: structural determinants to functions

Among the most important miRNAs, miR‐125b is implicated in a variety of cancers as either an oncogene or a suppressor. It is highly conserved in mammals, other vertebrates and nematodes.^24^ miR‐125b is the mammalian orthologue of *cel‐lin‐4*.^2^, ^3^ miR‐125b is very similar to the sequence of *cel‐lin‐4* with the differences located only in the central region, which is presumed to be bulged out during target mRNA recognition.^4^, ^7^ In humans, miR‐125b belongs to the miR‐125 family, which consists of three homologues, namely *hsa‐miR‐125a*, *hsa‐miR‐125b‐1* and *hsa‐miR‐125b‐2*.^25^ miR‐125b is transcribed from two loci located on chromosomes 11q23 (*hsa‐miR‐125b‐1*) and 21q21 (*hsa‐miR‐125b‐2*).^25^, ^26^ The two paralogues, *hsa‐miR‐125b‐1* and *hsa‐miR‐125b‐2*, code for the same mature sequence.^25^ In other species, there are different numbers of miR‐125b homologues in the genome. For example, in zebrafish there are three homologues (ie *dre‐miR‐125a*, *dre‐miR‐125b* and *dre‐miR‐125c*), whereas the genome contains only one homologue in chimpanzees (*ppa‐miR‐125b*), pufferfish (*tni‐miR‐125b*) or worms (*sme‐miR‐125b* or *sja‐miR‐125b*).^24^ Although the nomenclature differs, all the homologues share the same seed region.^24^

In humans, miR‐125b‐1 is organized in a cluster with miR‐100 and let‐7‐a.^25^ *Hsa‐miR‐125b‐1* is implicated in t(11;14)(q24;q32) and t(2;11)(p21;q23) chromosomal translocations, which leads to B‐cell acute lymphoid leukaemia (B‐ALL) and acute myeloid leukaemia (AML), respectively.^27^, ^28^, ^29^, ^30^ miR‐125b‐2 is in the second cluster along with miR‐99a and let‐7c.^25^ *Hsa‐miR‐125b‐2* lies ~50 kb downstream of both *hsa‐miR‐99a* and *hsa‐let‐7c*, which locate ~650 bp from each other. A growing number of studies on miR‐125b suggest that this particular miRNA plays an important role in various cellular processes, such as cell proliferation, apoptosis, differentiation and embryogenesis, by targeting many different proteins such as matrix metalloproteinases, transcription factors and growth factors.^31^, ^32^, ^33^, ^34^, ^35^, ^36^

## 2 MIR‐125b IN CANCERS

### 2.1 MiR‐125b is involved in various signalling pathways

The role of miR‐125b in the signalling pathways that underlie cancer development and progression has been explored comprehensively, and it is known to act largely through its multiple molecular targets, which are involved in signalling cascades, such as the canonical Wnt, PI3K/Akt, STAT‐3, MAPK, NF‐κB and p53 pathways.

#### 2.1.1 The Wnt and PI3K/Akt signalling pathways

**FIGURE 1:**
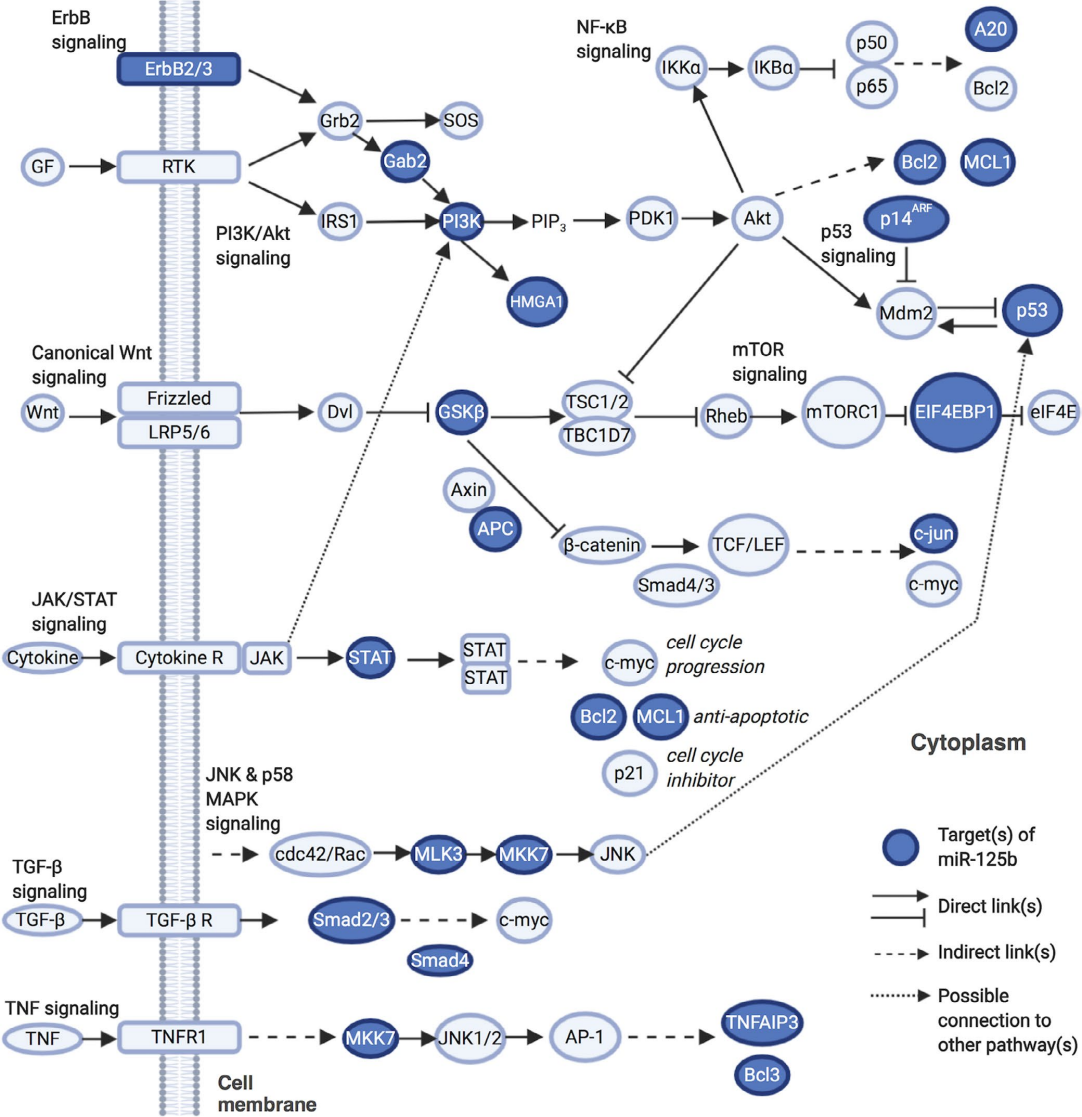
The signalling pathways mediated by miR‐125b. miR‐125b participates in many intracellular signalling pathways involved in the pathogenic process of cancer by directly regulating its target molecules (dark blue), which are key effectors within their cascades. The integrated signalling pathways and corresponding genes (light blue) are shown in the figure. *Created with* BioRender.com. GF, growth factor; GSK, glycogen synthase kinase; IKKα, IκB kinase α; IRS1, insulin receptor substrate 1; LEF, lymphoid enhancer‐biding factor; LRP, low‐density lipoprotein receptor‐related protein; PDK1, phosphoinositide‐dependent kinase 1; RTK, receptor tyrosine kinase; SOS, son of sevenless; TCF, transcription factor; TGFβ, transforming growth factor β; TNF, tumour necrosis factor; TNFAIP3, tumour necrosis factor α‐induced protein 3; TSC1/2, tuberous sclerosis 1/2

The Wnt/β‐catenin (canonical) signalling pathway is commonly activated in various types of cancer. miR‐125b regulates a series of targets associated with this pathway by exerting its function either as an oncogene or tumour suppressor (Figure 1). Oncogenic miR‐125b tends to up‐regulate Wnt/β‐catenin activity by down‐regulating targets responsible for the degradation of β‐catenin, thereby allowing more downstream targets such as proto‐oncogene *c‐MYC* to be expressed.^37^, ^38^ One of miR‐125b downstream targets is the tumour suppressor, adenomatous polyposis coli (APC), and loss‐of‐function mutations in APC have been linked to the progression of cancer.^39^ It has been shown, for example that in triple‐negative breast cancer, miR‐125b binds to the 3′ UTR of *APC*, and the suppression of miR‐125b activity results in decreased intracellular Wnt/β‐catenin signalling and EMT activity.^40^ The Wnt/β‐catenin pathway is also activated by a positive loop between the oncogenic CXCL12/CXCR4 axis and miR‐125b via APC in colorectal cancer.^41^ However, miR‐125b acts as a tumour suppressor in human papillomavirus (HPV) E6‐infected oesophageal cancer, as its overexpression reverses the down‐regulation of the tumour suppressor GSK3β in the H6‐up‐regulated Wnt signalling pathway.^42^ miR‐125b has also been shown to down‐regulate c‐Jun expression post‐transcriptionally in melanoma.^43^

Similarly, the dysregulated activation of the PI3K/Akt signalling pathway is commonly associated with the proliferative ability of tumour cells in cancer progression, and miR‐125b is also implicated in this signalling pathway (Figure 1). The overexpression miR‐125b decreases the levels of p‐PI3K and p‐AKT in bladder cancer.^44^ In addition, miR‐125b is up‐regulated in cervical cancer and it exerts its oncogenic function by targeting the 3′ UTR of high mobility group A (*HMGA1*), a tumour suppressor in the PI3K/Akt signalling pathway.^45^ In contrast, miR‐125b‐2 has been shown to be down‐regulated in gastric cancer cell lines and primary tissues. The exogenous expression of miR‐125b‐2 in gastric cancer cells has been shown to lead to decreased *PIK3CB* expression, thereby affecting the levels of oncogenic PI3Kβ in the PI3K/Akt pathway.^46^ In addition, in anaplastic thyroid cancer (ATC) cells, miR‐125b can affect the migration and invasion of ATC cells by directly targeting the phosphoinositide 3‐kinase catalytic subunit delta (PIK3CD) to repress PI3K, p‐Akt and p‐mTOR.^47^

The Wnt and PI3K/Akt signalling pathways are closely linked in cancer as they share common molecules such as GSK3β (Figure 1).^48^ It has been shown, for example that a combination of both PI3K and miR‐125b inhibitors causes inactivation of the Wnt signalling in temozolomide‐treated glioblastoma stem cells.^48^ In addition, EIF4EBP1, which plays a key role in the mTOR signalling pathway, downstream of Wnt and PI3K/Akt pathways, is post‐transcriptionally inactivated by miR‐125b in ovarian cancer.^49^

#### 2.1.2 STAT3‐related signalling pathways

STAT3 can be activated by various growth receptors and tumour‐associated factors, such as IL‐6, and it is a direct target of miR‐125b (Figure 1).^50^, ^51^ For example, the ectopic expression of miR‐125b in human osteosarcoma cells can reverse their high rate of proliferation, migration and tumour formation by targeting STAT3.^50^ In these cells, STAT3 regulates the level of miR‐125b by binding to its promoter region as a transactivator.^50^ STAT3 is also a direct target of miR‐125b in cervical cancer, and it also can be modulated by an upstream regulator of miR‐125b, called lncRNA SNHG12.^51^

miR‐125b is also implicated in the MAPK/STAT3 signalling pathway (Figure 1). MAP kinase kinase 7 (MKK7) activates c‐Jun N‐terminal kinase (JNK), which is linked to STAT3 phosphorylation, and it can be repressed by miR‐125b in osteosarcoma.^52^ For example, the down‐regulation of MKK7 by miR‐125b results in suppression of the proliferative ability and invasiveness of osteosarcoma cells in vitro and tumour formation in vivo.^52^ miR‐125b has also been shown to repress the expression of MAP2K7 and thus inhibit the EMT of Hs578T breast cancer cells.^53^ Furthermore, it exerts its oncogenic effects in B‐cell leukaemia by down‐regulating the expression of the tumour suppressor MAP3K11.^54^

miR‐125b exerts its effects on downstream targets, such as sphingosine‐1‐phosphate receptor 1 (S1PR1), which is a G protein‐coupled receptor that promotes tumourigenesis by activating p‐STAT3 directly.^55^ For example, the overexpression of miR‐125b‐1‐3p inhibits S1PR1 as well as the proliferative, invasive and migratory capability of non‐small cell lung cancer (NSCLC) cells.^56^

#### 2.1.3 NF‐κB pathway

miR‐125b is linked to the NF‐κB signalling pathway in cancer (Figure 1). The ectopic expression of miR‐125b in B‐cell lymphoma results in an enhanced NF‐κB activity, thereby facilitating cell proliferation and cancer progression in malignant B‐cell lymphoma.^56^ miR‐125b increases and maintains high NF‐κB activity via directly binding to the ubiquitin‐editing enzyme TNFAIP3 (tumour necrosis factor α‐induced protein 3).^57^ TNFAIP3 is also a direct target of miR‐125b in the NF‐κB signalling pathway in nasopharyngeal carcinoma.^57^ In nasopharyngeal carcinoma nude mouse model, the administration of miR‐125b antagomir inhibits cell proliferation and tumour growth.^58^

#### 2.1.4 p53 pathway

miR‐125b has also been implicated as a tumour suppressor in the p53 signalling pathway. For example, in glioblastoma cells, miR‐125b down‐regulates p53 and the knockdown of miR‐125b can lead to the activation of p53‐related apoptosis.^59^ Similarly, p53 expression is also down‐regulated in breast cancer cells infected with a retroviral vector containing miR‐125b.^60^ In addition, miR‐125b exerts its oncogenic effects by inhibiting p14^ARF^ in both LNCaP and 22Rv1 prostate cell lines as well as in prostate cancer xenograft mouse models.^61^ Subsequent treatment with an miR‐125b inhibitor results in an increase in p14^ARF^ and a decrease in Mdm2, as well as apoptosis via the p53 signalling pathway.^61^ miR‐125b is also found to suppress several different novel targets in the p53 pathway ranging from apoptotic regulators such as *Tp53*, to cell cycle regulators such as *Cdc25c*, as validated via gain‐of‐function, loss‐of‐function, pull‐down and luciferase assays.^33^

### 2.2 Upstream regulation of miR‐125b

A precise control of miR‐125b level is essential for maintaining the survival of human cells and for the development of zebrafish embryos.^31^, ^32^ The aberrant expression of miR‐125b has been demonstrated to give rise to a wide range of malignancies. Delineating the mechanisms and upstream regulators, such as transcription factors, lncRNAs, DNA methylation and histone modifications, which account for the altered expression of miR‐125b, will help researchers understand the important steps for modulation in the pathogenesis of cancer.

#### 2.2.1 Transcriptional regulation

**FIGURE 2:**
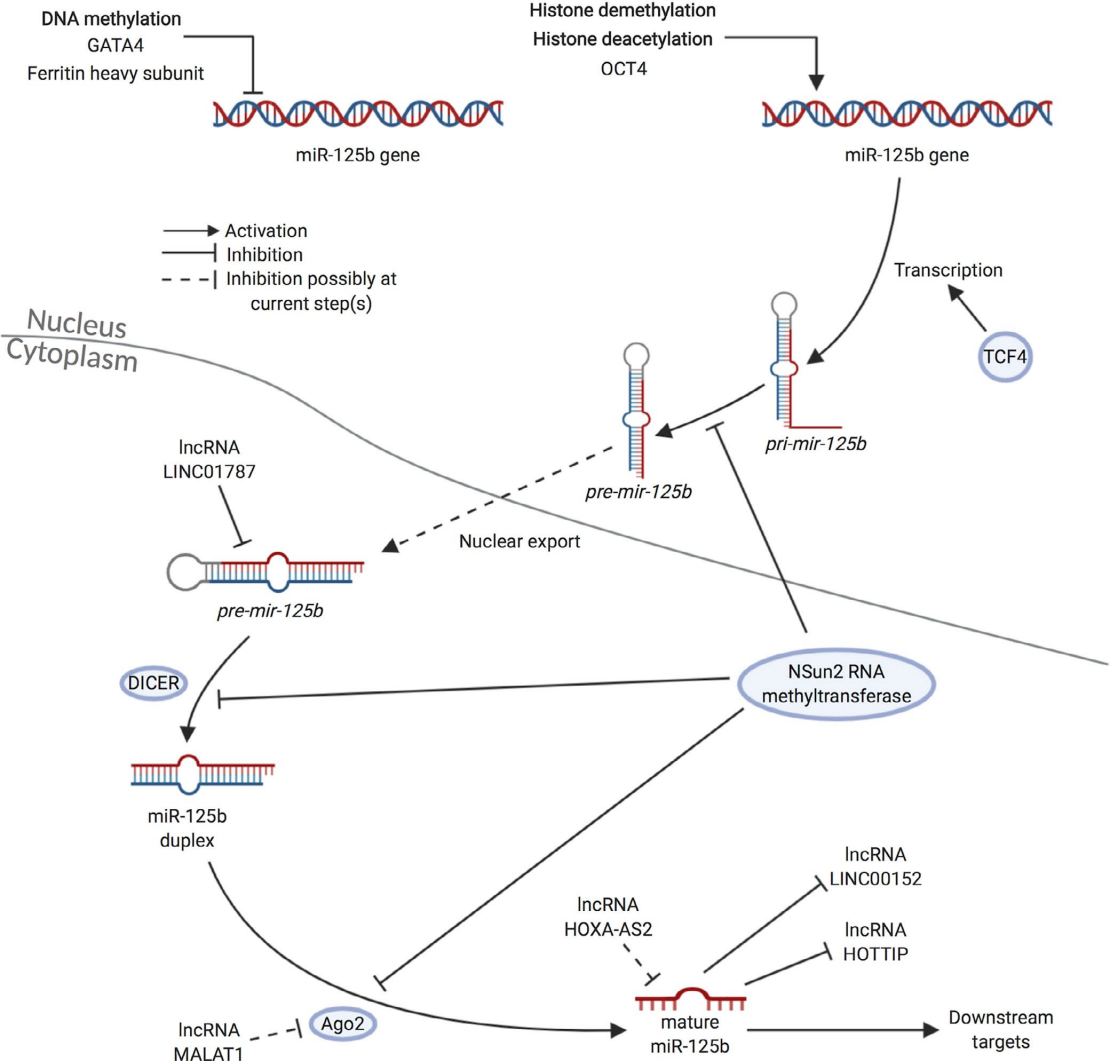
Upstream regulation of miR‐125b. There are multiple mechanisms underlying the regulation of miR‐125b by its upstream regulators during its biogenesis. These upstream regulations include transcription factor regulation (ie TCF4, OCT4, GATA4 and ferritin heavy subunit), lncRNA regulation (ie lncRNA LINC01787, lncRNA MALAT1, lncRNA HOXA‐AS2, lncRNA LINC00152 and lncRNA HOTTIP), RNA methylation (ie Nsun2 RNA methyltransferase), DNA methylation, histone (de)methylation and histone (de)acetylation, which account for the altered expression of miR‐125b. *Created with* BioRender.com. HOTTIP, HOXA distal transcript antisense RNA; HOXA‐AS2, HOXA cluster antisense RNA 2; MALAT1, metastasis‐associated lung adenocarcinoma transcript 1; OCT4, octamer‐binding transcription factor 4; TCF4, transcription factor 4

The zinc finger DNA‐binding protein, GATA4, has been shown to promote cancer progression in Huh6 human hepatoblastoma cells as it suppresses the expression of miR‐125b by binding to its promoter region (Figure 2).^62^ In addition, the ectopic overexpression of the heavy chain of ferritin, a nanocage protein, leads to the down‐regulation of miR‐125b in NSCLC via hypermethylation in the miR‐125b promoter region (Figure 2).^63^ In contrast, octamer‐binding transcription factor 4 (OCT4), which is involved in the maintenance of stemness in embryonic stem cells, promotes the expression of oncogenic miR‐125b in cervical cancer cells by directly binding to the miR‐125b promoter region, and this results in the suppression of the pro‐apoptotic protein, Bcl‐2 homologous antagonist/killer (BAK1) (Figure 2).^64^ In addition, transcription factor 4 (TCF4) contributes to the elevated migration and invasion observed in melanoma progression, again by promoting the expression of miR‐125b (Figure 2).^64^

#### 2.2.2 Long non‐coding RNA (LncRNA)

LncRNAs and miR‐125b are both non‐coding RNAs that have distinct characteristics with comparable regulatory functions in biological processes. LncRNAs are usually less than 200 nucleotides in length, and they play important regulatory roles at both the transcriptional and epigenetic levels.^65^ As such, they have been identified as upstream regulators and downstream targets of miR‐125b.

A variety of lncRNAs modulate expression of miR‐125b in cancers (Figure 2).^51^, ^66^, ^67^, ^68^ They are largely oncogenic as they down‐regulate the levels of miR‐125b. For example, lncRNA LINC01787 has been shown to drive the development of breast cancer as it binds to pre‐mir‐125b and thus prevents it from being processed by DICER.^66^ This results in a decrease in the levels of mature miR‐125b for downstream targeting. In addition, miR‐125b is down‐regulated by lncRNA HOXA cluster antisense RNA 2 (HOXA‐AS2) in bladder cancer.^67^ The subsequent knockdown of lncRNA HOXA‐AS2 inhibits the capability of bladder cancer cell lines to proliferate and migrate in vitro as well as tumour development in vivo.^67^ Moreover, the inhibitory effect of HOXA‐AS2 siRNAs on proliferation and migration of bladder cancer cells is attenuated by the addition of an miR‐125b inhibitor.^67^

Regarding the lncRNAs that are downstream targets of miR‐125b, the post‐transcriptional regulation of oncogenic lncRNA HOTTIP (HOXA distal transcript antisense RNA) is controlled by miR‐125b in liver cancer.^69^ Indeed, the ectopic expression of miR‐125b in hepatocellular carcinoma cells represses levels of lncRNA HOTTIP. miR‐125b is also known to target unregulated lncRNA LINC00152 in ovarian cancer.^70^

It has been suggested that there might be two‐way communication between lncRNAs and miR‐125b in some cancers. For example, in bladder cancer, metastasis‐associated lncRNA lung adenocarcinoma transcript 1 (MALAT1) has been identified as a downstream target of miR‐125b, but it is also known to suppress miR‐125b.^68^

In addition to lncRNAs, several other molecules that regulate miR‐125b expression have been identified.^71^, ^72^, ^73^, ^74^, ^75^, ^76^, ^77^

#### 2.2.3 DNA methylation

The expression of miR‐125b is modulated by DNA methylation of its promoter region as well as DNA methylation of the tumour suppressor genes that target it (Figure 2).^45^, ^46^, ^47^, ^48^ Higher methylation rates of miR‐125b contributing to down‐regulated level of miR‐125b have been discovered in colorectal tumour biopsies.^71^ In addition, the expression of miR‐125b is suppressed in nucleophosmin‐anaplastic lymphoma kinase (NPM‐ALK)^+^ systemic anaplastic large‐cell lymphoma, due to hypermethylation of the miR‐125b promoter region.^72^ NPM‐ALK activity along with DNA methyltransferase 1 (DNMT1) and DNA topoisomerase II (Topo II) is responsible for the hypermethylation of miR‐125b and thus the suppression in its level of expression. The subsequent inhibition of DNMT1 or Topo II leads to a reversal in the suppression of miR‐125b. Regarding the methylation of tumour suppressor genes, patients with promoter hypermethylation in genes such as *PTEN* and *RASSF1A* are also associated with down‐regulated miR‐125b levels.^73^

#### 2.2.4 Histone modifications

miR‐125b expression can also be regulated via both histone (de)methylation and (de)acetylation (Figure 2).^74^, ^75^, ^76^, ^77^ For example, KDM4B, which is a frequently up‐regulated histone demethylase, activates the Wnt signalling by up‐regulating miR‐125b in gastric cancer.^75^ In addition, the enhanced methylation of histones H3K9me3 and H3K27me3 is associated with the down‐regulation of miR‐125b‐1 in breast cancer cells.^77^ Subsequent treatment with EZH2, a H3K27 histone methyltransferase inhibitor, increases the expression of miR‐125b‐1.^77^ Furthermore, treatment with entinostat, a class I histone deacetylase (HDAC) inhibitor, up‐regulates the miR‐125b levels in erbB2/erbB3‐positive breast cancer cells.^76^

### 2.3 MiR‐125b in cancer biology

The development of cancer is a multi‐step process involving multiple alterations in oncogenes and tumour suppressor genes over time. A large number of studies have established an important role for miR‐125b in the pathogenesis of cancer. It can function either as an oncomiR in immunomodulation and intercellular communications or as a tumour suppressor in regulating glycosis, or it can exert both oncogenic and tumour‐suppressive effects on processes such as apoptosis and metastasis.

#### 2.3.1 Glycolysis regulation

The activity of miR‐125b is linked to glycolysis in cancer (Table 1). Hexokinase‐2 (HK‐2) is an enzyme involved in catalysing the first rate‐limiting step in glycolysis, and it is targeted by miR‐125b‐5p.^44^, ^78^ In laryngeal squamous cell carcinoma (LSCC) cells, miR‐125b‐5p overexpression leads to the down‐regulation of HK‐2 and thus results in a decrease in lactate production and glucose consumption.^78^ The overexpression of miR‐125b also results in increased apoptosis and decreased proliferation in LSCC cells. HK‐2 is associated with the PI3K/Akt signalling pathway and with miR‐125b overexpression in bladder cancer.^44^ ErbB2 is also a glycolytic target of miR‐125b.^79^ miR‐125b overexpression suppressed ErbB2‐mediated glycolysis; thus, it sensitizes both parental and doxorubicin‐resistant chondrosarcoma cells to doxorubicin treatment.^79^

**TABLE 1:** Summary of the role of miR‐125b in cancer progression

Type of cancer	Expression	Target(s)	Function
LSCC	↑	HK‐2^78^	Glycolysis↓ Apoptosis↑ Proliferation↓
Bladder cancer	↑	HK‐2^44^	Cell viability↑ Migration↑ Apoptosis↓
Chondrosarcoma	↑	ErbB2^79^	Glycolysis↓ Chemosensitivity↑
Lung cancer	↑	BAX^37^	Apoptosis↓
		TP53INP1^80^	Metastasis↑
	↓	MMP‐13,^81^ KLC2^82^	Invasion↑ Migration↑
	Ex‐↑		Chemosensitivity↑^83^
Ewing's sarcoma	↑	BAK1,^84^ p53^74^	Chemoresistance↑
Myeloid leukaemia	↑	BAK1^85^	Proliferation↑ Apoptosis↓
Osteosarcoma	↓	Bcl‐2^86^	Proliferation↑ Migration↑ Invasion↑ Chemosensitivity↓
Ovarian cancer	↓	MCL‐1^70^	Apoptosis↑
		BCL3^87^	Proliferation↑ Tumour formation↑
		Gab2^88^	Migration↓
		SET^89^	Invasion↓ EMT↓
	Ex‐↑		Diagnosis↑^90^
Cervical cancer	↑		CSC property↑^64^ Proliferation↓^64^ Tumourigenesis↑
Hepatocellular carcinoma	↓	SIRT6^91^	Senescence↑ Apoptosis↑
		SIRT7^92^	Proliferation↓
		Ang2,^93^ TXNRD1^94^ Ang2^93^	Metastasis↓ VETC formation↓
	Ex‐↑		Diagnosis↑^95^ Recurrence↓^96^ Overall survival↑^96^
Breast cancer	↑	STARD13,^97^TP53INP1^98^	Metastasis↑
		SAF‐1^99^	VEGF activity↓ Proliferation↓ Invasion↓
	Ex‐↑	TP53INP1,^100^ TP53^100^	CAFs activation↑
Melanoma	↑	NEDD9^101^	Metastasis↑
Glioma	↑		Invasion↓^89^
		E2F2^102^	Proliferation↓
		MMP‐9^103^	CSC property↑
	Ex‐ ↓		Diagnosis↑^104^
T‐ALL	↑	Ets1,^105^ CBFβ^105^	HSC property↑ T progenitor production↑ T‐cell differentiation↓ Cancer development↑
		A20,^106^ NF‐κB^106^	CD4^－^ T‐cell production↑ Glucose metabolism↑
Megakaryocytic leukaemia	↑	GATA1^107^	HSC property↑ Proliferation↑
B‐cell lymphoma	↑	S1PR1,^108^ IRF4^108^	HSC property↑ B‐cell development↓
Colorectal cancer	↓	MMPs^109^	Invasion↑ Migration↑
	Ex‐↑		Diagnosis↑^110^ Chemosensitivity↓^111^

Abbreviations: ↑, up‐regulation; ↓, down‐regulation; Ang2, angiopoietin 2; BAK1, Bcl‐2 homologous antagonist/killer; BAX, Bcl‐2 associated X protein; Bcl‐2, B‐cell lymphoma 2; CAFs, cancer‐associated fibroblasts; CSC, cancer stem cell; EMT, epithelial–mesenchymal transition; Ex‐, extracellular expression; HK‐2, Hexokinase‐2; HSC, haematopoietic stem cell; KLC2, Kinesin‐1 light chain‐2; LSCC, laryngeal squamous cell carcinoma; MCL‐1, myeloid leukaemia cell differentiation protein 1; MMP‐13, metalloproteinase 13; MMPs, metalloproteinases; NEDD9, neural precursor cell expressed developmentally down‐regulated protein 9; S1PR1, sphingosine‐1‐phosphate receptor 1; SAF‐1, serum amyloid A‐activating factor 1; SIRT6, Sirtuins 6; SIRT7, Sirtuins 7; STARD13, StAR‐related lipid transfer domain protein 13; T‐ALL, T‐cell acute lymphoblastic leukaemia; TP53, tumour protein p53; TP53INP1, tumour protein p53‐inducible nuclear protein 1; TXNRD1, thioredoxin reductase 1; VEGF, vascular endothelial growth factor; VETC, vessels that encapsulated tumour clusters.

#### 2.3.2 Apoptosis regulation

miR‐125b exerts its oncogenic and tumour‐suppressive functions by targeting pro‐apoptotic and anti‐apoptotic proteins (Table 1). miR‐125b controls the expression of pro‐apoptotic genes, such as *BAK* and *p53*. The down‐regulation of pro‐apoptotic proteins is linked to cancer proliferation and progression. For example, the down‐regulation miR‐125b promotes the expression of Bcl‐2‐associated X protein (BAX) in NSCLC A549 cells.^37^ It also has been shown to target *BAK* and *p53* directly in Ewing sarcoma/primitive neuro‐ectodermal tumour,^84^ and it contributes to the increased proliferation and decreased apoptosis observed in chronic myeloid leukaemia, by repressing the activity of BAK1.^85^

miR‐125b regulates the expression of anti‐apoptotic genes such as B‐cell lymphoma 2 (*Bcl‐2*). The up‐regulation of anti‐apoptotic proteins is also linked to cancer progression (Table 1). For example, miR‐125b down‐regulates *Bcl‐2* in osteosarcoma, thereby sensitizing osteosarcoma cells to cisplastin treatment.^86^ miR‐125b also targets myeloid leukaemia cell differentiation protein 1 (MCL‐1), which is an anti‐apoptotic family member of Bcl‐2. MCL‐1 expression is also mediated by LINC00152, and both have been shown to share the same miR‐125b combining sites in ovarian cancer.^70^ In addition, the ectopic expression of miR‐125b inhibited the translation of *BCL3* mRNA, and thus contributes to the decreased proliferation and tumour formation in ovarian cancer.^87^

miR‐125b is involved in the regulation of sirtuins (SIRTs; Table 1). SIRTs are class III deacetylases, and they are involved in various cellular processes, including several that are involved in cancer tumourigenesis.^91^ SIRT6 activity can be directly inhibited by miR‐125b via targeting the seed‐matching region in the 3′ UTR.^91^ miR‐125b overexpression or SIRT6 knockout leads to cellular senescence and apoptosis in hepatocellular carcinoma cells.^91^ Similarly, SIRT7 activity is directly repressed by miR‐125b in hepatocellular carcinoma HepG2 cells.^92^

#### 2.3.3 Metastasis regulation

Metastasis refers to the spread of cancer cells from the site of origin to distant sites to form secondary tumours, known as metastases. Several molecules that promote or inhibit metastasis, which are associated with miR‐125b, have been identified (Table 1). miR‐125b has been shown to elevate the metastatic activity of NSCLC by repressing metastasis inhibitor, tumour protein p53‐induced nuclear protein 1 (TP53INP1).^80^ In NSCLC metastasis, miR‐125b exerts its tumour‐suppressive effects when elevated levels decrease the expression of metalloproteinase 13 (MMP‐13) and down‐regulate the invasive ability of the cancer cells.^81^ Similarly, miR‐125b exerts its suppressive effects on NSCLC cell invasion and migration by directly targeting kinesin‐1 light chain‐2 (KLC2) protein.^82^ miR‐125b exerts its pro‐metastatic potential in breast cancer cells by directly targeting tumour suppressor StAR‐related lipid transfer domain protein 13 (STARD13).^97^ It is also known that TP53INP1 and STARD13 have competitive endogenous RNA (ceRNA) interactions as both competitively bind to miR‐125b in the metastatic regulation of breast cancer.^98^ miR‐125b is strongly expressed in metastatic melanomas, and its direct downstream target, neural precursor cell expressed developmentally down‐regulated protein 9 (NEDD9), is decreased at both the mRNA and protein levels.^101^ The repression of miR‐125b abolishes the cell migration induced by ovarian cancer ascites via regulating *Gab2*.^88^ Ovarian cancer cell migration and invasion are inhibited when miR‐125b binds directly to the *SET* 3′ UTR both in vitro and in vivo, which results in the suppression of EMT.^112^ In addition, the overexpression of miR‐125b decreases the invasive properties of paediatric low‐grade glioma‐derived cells.^89^ Furthermore, it also attenuates hepatocellular carcinoma metastasis by suppressing vessels that encapsulate tumour clusters (VETC), by targeting angiopoietin 2 (Ang2).^93^ Another identified target of miR‐125b in regulating hepatocellular carcinoma metastasis is thioredoxin reductase 1 (TXNRD1), a regulator of endocellular oxidative stress and a promoter of tumour development.^94^ miR‐125b can also negatively regulate cell invasion and migration as well as the activity of metalloproteinases (MMPs) in metastatic colorectal cancer cells with submucosal invasion.^109^

Angiogenesis also plays a part in cancer metastasis, and vascular endothelial growth factor (VEGF) is an important protein involved in this process. Serum amyloid A‐activating factor 1 (SAF‐1) elevates the activity of VEGF and is a downstream target of miR‐125b. The ectopic expression of miR‐125b has been shown to inhibit SAF‐1 expression, and thus down‐regulate the proliferative and invasive nature of breast cancer cells (Table 1).^99^

#### 2.3.4 Cancer stem cell regulation

Cancer stem cells (CSCs) are a small subpopulation of cells within tumours with properties of self‐renewal, differentiation and tumourigenicity when transplanted into an animal host.^113^ miR‐125b levels are found to be regulating the self‐renewal and differentiation capabilities of CSCs. For instance, miR‐125b overexpression is found to suppress proliferative ability and CD133 stem cell marker of CD133‐positive glioma cancer stem cells via inhibition of *E2F2*.^102^, ^114^, ^115^ Up‐regulated miR‐125b levels are found to suppress proliferation via cell cycle arrest at G_1_ to S transition, coupled with decreased levels of cell cycle regulated proteins CDK6 and CDC25A.^114^ On the other hand, miR‐125b is associated with oncogenic properties as it is highly expressed in highly invasive glioma stem cell and progenitor (GSCP) cell lines, SU3 and SU2 with possible association to MMP9 levels.^103^ Also, octamer‐binding transcription factor 4 (OCT4), an important regulator gene involved in the maintenance of self‐renewal properties in stem cells, was also found to bind to miR‐125b‐1 to increase miR‐125b levels, thereby suppressing apoptosis and promoting tumourigenesis in cervical cancer.^64^

Haematopoietic stem cells are generally considered the base of the adult haematopoietic system, having the crucial function of long‐term maintenance and production of all mature blood cell lineages during the lifespan of an organism.^116^ A number of investigations have reported that the deregulation of miR‐125b influences critical developmental checkpoints during haematopoiesis and thus leads to the development of cancer (Table 1).^105^, ^106^, ^107^, ^108^ T‐cell acute lymphoblastic leukaemia (T‐ALL) is an aggressive haematopoietic malignancy with a 5‐year survival rate of ~50%.^117^ miR‐125b is up‐regulated in human T‐ALL.^105^, ^106^ Normally, miR‐125b is expressed in most bone marrow myeloid cells and multipotent haematopoietic stem cells.^118^ When miR‐125b is overexpressed in human haematopoietic progenitor cells, the production of T‐cell progenitors is enhanced, whereas the differentiation of immature T cells is blocked.^105^ In addition, the ectopic expression of miR‐125b contributes to the development of leukaemia and accelerates the maturation arrest in T‐ALL xenograft mouse models in part via the inhibition of the T‐lineage regulators, Ets1 and CBFβ.^105^ A similar investigation revealed that the overexpression of miR‐125b increases undifferentiated CD4^–^ cells in leukaemic T cells and regulates the reprogramming of glucose consumption in T cells by targeting A20, which is an important protein in the negative feedback inhibition of NF‐κB activation.^106^ In acute megakaryocytic leukaemia, miR‐125b is found to be ectopically expressed and target GATA1, which results in enhanced size and number of magakaryocytes and megakaryocytic erythorid progenitor colonies, thereby promoting proliferation of AMKL cell lines.^107^

miR‐125b is epigenetically silenced during the development of normal B cells.^108^ Indeed, it has been reported that the genes encoding miR‐125b‐1/2 lack transcriptional activating modifications and RNA polymerase II or p300 enrichment in the promoter region in mouse splenic resting B cells, pro‐B cells, pre‐B cells and B cells activated by LPS and IL‐4.^108^ In addition, dysregulated expression of miR‐125b initially reduces the production of B cells in the blood and spleen, and of pre‐B cells in the bone marrow, by targeting S1PR1, but later promotes pre‐B‐cell lymphoma via the inhibition of IRF4 (Table 1).^108^ Besides that, miR‐125b overexpression increases the number of early B‐progenitor cells and induces the expansion and enrichment of the lymphoid‐balanced and lymphoid‐biased haematopoietic stem cell subset via negatively regulating pro‐apoptotic targets, *Bmf* and *KLF3*, thereby conferring lymphoproliferative neoplasm.^119^

#### 2.3.5 Intercellular communication

The role of circulating miRNAs in carcinogenesis has garnered considerable scientific interest. Circulating miRNAs are increasingly recognized as being promising biomarkers, and they have become an attractive tool for liquid biopsies in cancer screening. Circulating miR‐125b contributes actively to cancer development and progression, making it a potential target for cancer prevention and therapy (Table 1). For instance, patients with HBV‐associated hepatocellular carcinoma have a higher level of miR‐125b in the serum than healthy donors.^95^ It has also been shown that the combination of serum miR‐27a and miR‐125b, in conjugation with α‐fetoprotein (AFP), can be used to enhance the specificity and sensitivity of AFP‐negative HBV‐associated early‐stage hepatocellular carcinoma diagnosis.^120^ An elevation of serum/plasma miR‐125b was identified as a diagnostic biomarker in patients with early colorectal neoplasms, epithelial ovarian cancer in early stages I and II, and pancreatic cancer, whereas a reduction in serum miR‐125b was observed in glioma patients.^90^, ^104^, ^110^, ^121^ Another group detected urinary miRNAs in patients with breast cancer and found that urinary miR‐125b is significantly lower than in healthy donors.^122^ In addition, lung cancer patients exhibit increased levels of plasma miR‐125b after chemotherapy and again after surgery as compared to untreated patients.^83^ Indeed, the miR‐125b/miR‐19b ratio undergoes dynamic changes during the course of chemotherapy, which suggests that circulating miR‐125b also acts as a predictive biomarker in the response to anti‐tumour therapy.^83^

Extracellular vesicles (EVs) are membranous nanoparticles that are secreted by all cell types.^123^ Depending on where they are formed, EVs can differentiate into exosomes or ectosomes. The former originate from the endocytic pathway and are released upon fusion of multivesicular bodies with the cell membrane, whereas the latter directly bud off from the cell membrane.^124^ EVs carry bioactive proteins, DNAs, RNAs (ie miRNAs, mRNAs and lncRNAs) and lipids that can be transferred to other cells thereby changing their physiological and pathological functions.^125^ Our study on breast cancer cell‐derived EVs demonstrated that miR‐125b can be transferred from cancer cells to resident fibroblasts within the tumour microenvironment. These fibroblasts were then differentiated into cancer‐associated fibroblasts (CAFs), which contribute to cancer progression and metastasis by suppressing *TP53INP1* in both mice and humans, as well as *TP53* in humans alone (Table 1).^100^ EVs are circulating freely in biological fluids, such as plasma, urine and cell conditioned medium, and they are therefore considered to be potential ‘liquid biopsies’, enabling non‐metastatic diagnosis and real‐time cancer monitoring (Table 1).^126^ In hepatocellular carcinoma patients, a remarkably high level of serum exosomal miR‐125b is detected; this results in a longer recurrence time and overall survival.^96^ In addition, a study of the plasma exosomal miRNAs in the response to mFOLFOX6 chemotherapy revealed that the plasma exosomal miR‐125b is increased in patients with progressive colorectal cancer as compared to those with stable colorectal cancer or no cancer.^111^ Furthermore, exosomal miR‐125b levels changed after patients with progressive cancer were treated with mFOLFOX6, suggesting that miR‐125b might facilitate the early screening of patients exhibiting mFOLFOX6 resistance.^111^

### 2.4 MiR‐125b in cancer treatment

#### 2.4.1 Therapeutics targeting miR‐125b

miR‐125b has been identified as a potential target in the development of anti‐cancer therapies due to its involvement in the different signalling pathways. In particular, the treatment with calcitriol and tacalcitol has been shown to cause a decrease in miR‐125b as well as an increase in pro‐apoptotic protein BAK1 in MCF‐7 breast cancer cells.^127^ In addition, there have been several studies reporting the use of nano‐therapeutic systems to encapsulate drugs targeting miR‐125b for cancer treatment. Silibinin, a therapeutic drug that is stably encapsulated in polymersome nanoparticles, suppresses oncogenic miR‐125b activity and thus overexpresses pro‐apoptotic proteins such as BAX in breast cancer cells.^128^ Hyaluronic acid‐based nanoparticles encapsulated with miR‐125b have also been engineered to target tumour‐associated macrophages, thereby facilitating the effectiveness of paclitaxel in treating ovarian cancer mouse models.^129^ Furthermore, the systemic delivery of both miR‐125b and p53 via CD44/EGFR‐targeted hyaluronic acid‐based nanoparticles leads to apoptosis and suppressed tumour growth in KrasG12D/p53fl/fl lung cancer mouse models.^130^ Remarkably, the delivery of miR‐125b ASOs using red blood cell extracellular vesicles to leukaemia MOLM13 cells and breast cancer MCF10CA1a cells results in reduced cell proliferation as the suppressed expression of miR‐125b can lead to an increased level of BAK1.^131^

Anti‐cancer immune responses help to prevent tumour outgrowth after conventional chemotherapy. Several studies have reported that many chemotherapeutic agents not only induce cancer cell death but also trigger immune stimulatory side effects. The bromodomain and extra‐terminal (BET) proteins play a critical role in regulating the transcription of oncogenes and the expression of osteoclastogenic cytokines.^132^ The treatment of multiple myeloma (MM) cell lines and patient‐derived CD138^+^ MM cells with BET inhibitors and ARV‐825 results in an increase in MICA (a ligand of the NK‐activating receptor). This renders the MM cells more efficient at activating NK‐cell degranulation.^132^ In addition, an inhibition of c‐MYC correlates with a decrease in its target gene, IRF4, and a subsequently increase in miR‐125b‐5p.^132^ A high level of expression of miR‐125b‐1 in colorectal cancer cells is associated with poor prognosis in HLA‐matched patients who have undergone peptide vaccination.^133^ In this case, miR‐125b‐1 works as an immune suppressor by negatively regulating RANK (receptor activator of NF‐κB).^133^ These data provide new insights on the immunomodulatory anti‐cancer activities targeting miR‐125b.

#### 2.4.2 Therapeutic resistance

**FIGURE 3:**
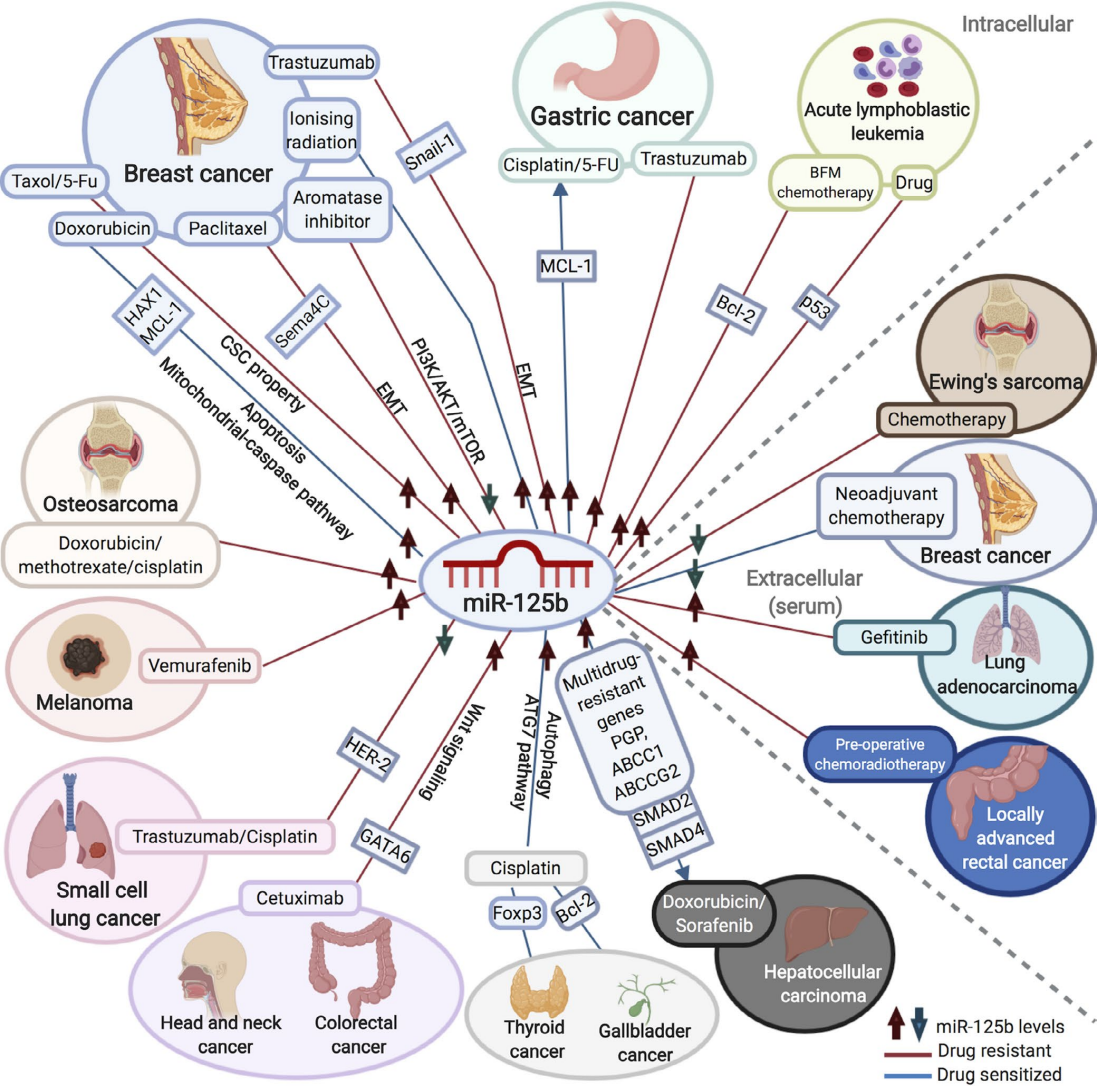
The association of miR‐125b in therapeutic resistance. Intracellular and extracellular miR‐125b plays an important role in regulating therapeutic resistance in cancer. The differential expression of miR‐125b renders different cancers distinct sensitivities to therapies by regulating its multiple molecular targets as well as various cellular processes and signalling pathways, which are shown in the figure. It even exhibits opposite regulatory properties in one type of cancer (ie breast cancer and gastric cancer). This indicates the complexity of therapeutic resistance mechanisms. *Created with* BioRender.com. ↑: up‐regulation; ↓: down‐regulation; CSC: cancer stem cell; EMT: epithelial–mesenchymal transition

Chemotherapy resistance is a critical problem in cancer treatment. Although many therapies are available for treating cancer, their ability to halt the progression of the disease remains limited due to the drug resistance mechanisms that cancer cells might develop.^134^ Failure of first‐line therapies often reduces the survival rate and leads to metastasis in many patients.^135^ Recent studies identified miR‐125b as both an intracellular and extracellular biomarker for chemotherapy resistance in many cancers (Figure 3).^136^, ^137^, ^138^, ^139^, ^140^, ^141^, ^142^, ^143^, ^144^, ^145^, ^146^, ^147^, ^148^, ^149^, ^150^, ^151^, ^152^, ^153^, ^154^, ^155^, ^156^, ^157^, ^158^, ^159^, ^160^ For example, two independent groups demonstrated that miR‐125b is down‐regulated in doxorubicin (DOX)‐resistant human breast cancer cell line MCF‐7 (MCF‐7/DR) as compared with the parental cell line and that the enforced expression of miR‐125b resensitizes these cells to DOX treatment.^136^, ^137^ They showed that miR‐125b increases apoptosis of MCF‐7/DR cells by targeting HAX‐1 or MCL‐1 through the mitochondrial‐caspase pathway.^136^, ^137^ Similarly, miR‐125b is down‐regulated in paclitaxel‐resistant MCF‐7 and SKR cells as well as in aromatase inhibitor (AI)‐resistant MCF‐7 cells.^138^, ^139^ In this case, miR‐125b activates the PI3K/AKT/mTOR pathway in AI‐resistant ER‐positive breast cancer cells and induces EMT via targeting *Sema4C* in the paclitaxel‐resistant cells.^138^, ^139^ It has also been shown that miR‐125b, when epigenetically modulated by lncRNA TINCR, regulates EMT via targeting *Snail‐1* in trastuzumab‐resistant HER2‐positive SKBR‐3 and BT474 breast cancer cells.^140^ In addition, up‐regulation of miR‐125b levels via Snail‐activated Wnt/β‐catenin‐TCF4 pathway in Snail‐overexpressing breast cancer cells is found to increase cancer stem cell population and confer Taxol chemoresistance during cancer treatment.^154^ miR‐125b overexpression is also found to be associated with the increase in stem cell‐like side population, thereby conferring 5‐FU chemoresistance in breast cancer.^155^ Studies on peripheral blood and bone marrow specimens from paediatric patients with acute lymphoblastic leukaemia (ALL) demonstrated that following Berlin‐Frankfurt‐Munster (BFM) chemotherapy, an elevation in miR‐125b and down‐regulation of *Bcl‐2* correlate with short leukaemia‐free survival.^141^, ^142^ Furthermore, miR‐125b is up‐regulated while its target p53 is down‐regulated in drug‐resistant *BCR‐ABL^+^* ALL.^143^ These data suggest the prognostic value of miR‐125b in predicting the resistance of patients to BFM chemotherapy. In gastric cancer, the overexpression of miR‐125b enhances the chemosensitivity of MGC‐803 and HGC‐27 cells to cisplatin, as well as of MGC‐803 cells to 5‐FU, via the down‐regulation of MCL1.^144^, ^145^ In contrast, an increased level of miR‐125b is associated with trastuzumab resistance in HER2‐positive gastric cancer.^146^ In other types of cancer, the differential expression patterns of miR‐125b elicit distinct sensitivities to chemotherapy. For example, the enhanced expression of miR‐125b in hepatocellular carcinoma cells reduces doxorubicin/sorafenib resistance by negatively regulating both *SMAD2* and *SMAD4*, as well as multidrug resistance genes, such as *PGP*, *ABCC1* and *ABCCG2*.^147^ In addition, an overexpression of lncRNA MIR100HG‐derived miR‐125b has been observed in cetuximab‐resistant colorectal cancer cells, head and neck squamous cell cancer cells and colorectal cancer patients by repressing GATA6 and thus activating the Wnt/β‐catenin signalling pathway.^148^ miR‐125b overexpression also induces resistance to methotrexate, doxorubicin and cisplatin in osteosarcoma cell lines.^149^ The ectopic expression of miR‐125b is induced by CCL2 in vemurafenib‐resistant melanoma cell lines and tumour biopsies.^150^ An up‐regulation of miR‐125b has been demonstrated to sensitize thyroid cancer cells and gallbladder cancer cells to cisplatin treatment by targeting *Foxp3* via the ATG7 pathway and by down‐regulating *Bcl‐2*, respectively.^151^, ^152^ In addition, trastuzumab has been shown to induce the overexpression of HER2 in HER2‐positive small cell lung cancer (SCLC) cells through the down‐regulation of both miR‐125b and miR‐125a.^153^ Cisplatin treatment also results in a decrease in the expression of miR‐125b and miR‐125a in SCLC.^153^ These data suggest the potentiality of miR‐125b replacement therapy for drug resistance prevention in HER2^+^ SCLC.

An increasing number of studies have also revealed the essential role of circulating miR‐125b in chemotherapy (Figure 3). For example, in stage II/III breast cancer patients, the level of serum miR‐125b has been found down‐regulated in neoadjuvant chemotherapy responders when compared with non‐responders, resulting in better disease‐free survival.^156^ The level of serum miR‐125b is also reduced in Ewing's sarcoma patients exhibiting poor response to chemotherapy.^157^ In addition, in human lung adenocarcinoma cells, a higher expression level of miR‐125b has been found in A549 and H1299 cells, which are gefitinib resistant, than in PC‐9 cells, which are sensitive to gefitinib.^158^ Furthermore, miRNA expression microarray analysis indicated that miR‐125b is up‐regulated in patient tissue samples containing EGFR mutation for gefitinib resistance as compared with samples containing EGFR mutation for gefitinib sensitivity.^158^ Thus, the overexpression of plasma miR‐125b in patients appears to lead to lower disease‐free survival.^158^

Radioresistance also poses a major challenge in cancer treatment. A significant number of patients suffer from locoregional recurrence due to the outgrowth of radio‐resistant cells.^161^ The forced expression of miR‐125b in MDA‐MB‐231 and MCF‐7 breast cancer cells induces sensitivity to ionizing radiation treatment, as seen by a decrease in the level of clonogenic survival and an increase in apoptosis as well as senescence after radiation, which is abrogated by the re‐expression of c‐Jun (Figure 3).^159^ A study on cancer biopsies and serum samples from locally advanced rectal cancer (LARC) patients involving pre‐operative chemo‐radiotherapy revealed that cellular and serum miR‐125b levels are elevated in non‐responders when compared with responders. This suggests that high tissue and serum miR‐125b levels are associated with poor treatment response in LARC patients (Figure 3).^160^

## 3 PROSPECTS

The discovery of miRNAs has broadened the understanding of human diseases, including cancer. In this paper, we have reviewed the essential functions of miR‐125b in tumourigenesis, cancer progression and cancer treatment. miR‐125b acts as an oncogene in breast cancer, haematopoietic malignancies, laryngeal squamous cell carcinoma, bladder cancer, sarcoma, chondrosarcoma, glioblastoma, nasopharyngeal carcinoma, prostate cancer, cervical cancer, thyroid cancer, melanoma and glioma, while as a tumour suppressor in osteosarcoma, ovarian cancer, oesophageal cancer, gastric cancer, hepatocellular carcinoma and colorectal cancer. Notably, miR‐125b plays a controversial role in lung cancer. miR‐125b is regulated by multiple factors, and the interaction of miR‐125b with its targets constitutes their regulatory network. The complexity of the network provides miR‐125b with a wide range of biological functions, depending on the cellular context. This network also ensures that miR‐125b has great potential in clinical application. miR‐125b represents a potential diagnostic and prognostic biomarker, and it can also be considered as a potential therapeutic target in cancer treatment even though it has different regulatory functions in chemoresistance in different cancers. Therefore, a more detailed understanding of mechanisms underlying how miR‐125b regulates therapeutic resistance might be a focus in the future. Given the dual functions of miR‐125b in cancers, it is necessary to adopt different analyses and interference strategies in different cancers. Taking into account the fact that miR‐125b can effectively regulate the response of cancer cells to chemotherapy, it is promising to use a combination of miR‐125b‐targeted treatments with chemotherapy in certain types of cancer to achieve a better therapeutic effect.

## CONFLICTS OF INTEREST

ML is the cofounder of Carmine Therapeutics, an extracellular vesicle company. Other authors have no conflict of interest.

## AUTHOR CONTRIBUTIONS

ML, PB and TPY conceptualized the review. PB and TPY wrote the manuscript. ML critically reviewed and edited the manuscript. All authors read and approved the final manuscript.

## Data Availability

The data that support the findings of this study are available from the corresponding author upon reasonable request.
